# Supplementary material for: Hybrid Models and Biological Model Reduction with PyDSTool
Source: PLoS Comput Biol. 2012 Aug 9;8(8):e1002628. doi: 10.1371/journal.pcbi.1002628 (PMC3415397; doi:10.1371/journal.pcbi.1002628)
Supplement: Text S4 — Complete source code for the PyDSTool package (version 0.88.120504). Includes API documentation and help files linking to web pages. This file is identical to the current public release on Sourceforge.net. (ZIP) [file pcbi.1002628.s004.zip › PyDSTool/html/toc-PyDSTool.Toolbox.optimizers.defaults-module.html]

xml version="1.0" encoding="ascii"?


defaults


# Module defaults

---

## Variables

FAILED\_WITH\_UNIMPLEMENTED\_OR\_UNKNOWN\_REASON

FVAL\_IS\_ENOUGH

IS\_ALL\_VARS\_FIXED

IS\_LINE\_SEARCH\_FAILED

IS\_MAX\_CPU\_TIME\_REACHED

IS\_MAX\_FUN\_EVALS\_REACHED

IS\_MAX\_ITER\_REACHED

IS\_MAX\_TIME\_REACHED

IS\_NAN\_IN\_X

SMALL\_DELTA\_F

SMALL\_DELTA\_F\_F

SMALL\_DELTA\_X

SMALL\_DELTA\_X\_X

SMALL\_DF

SOLVED\_WITH\_UNIMPLEMENTED\_OR\_UNKNOWN\_REASON

errors  
 parameters  

---

[hide private]
